# Esophagitis Dissecans Superficialis: A Frequently Missed and Rarely Reported Diagnosis

**DOI:** 10.7759/cureus.21647

**Published:** 2022-01-26

**Authors:** Godson Senyondo, Ali Khan, Fahad Malik, Amanke Oranu

**Affiliations:** 1 Department of Gastroenterology, Wilson Medical Center, Johnson City, USA

**Keywords:** ppi, parakeratosis, mucosal sloughing, minimal inflammation, esophagitis

## Abstract

Esophagitis dissecans superficialis (EDS) is a rare esophageal disease with a wide spectrum of presentations from asymptomatic to debilitating symptoms. There is a strong association of EDS with autoimmune diseases, smoking, and medications, but it can also be idiopathic. Due to the sporadic occurrence of EDS, identification requires a high index of suspicion to avoid frequent misdiagnoses. Herein, we present a case of EDS associated with the long-standing use of oral diclofenac with a favorable outcome after therapy with a proton pump inhibitor (PPI).

## Introduction

Esophagitis dissecans superficialis (EDS) is a rare, benign endoscopic finding, characterized by mucosal sloughing into the esophageal lumen [1]. The etiology of EDS is currently unclear. While some cases are idiopathic, others are linked to mucosal irritants such as medications, smoking and alcohol, hot beverages, and bullous skin conditions [2-3]. The diagnosis of EDS is frequently missed, as the histopathology is underrecognized due to sample contamination. We report a case of EDS in a 54-year-old male on chronic oral diclofenac who presented with epigastric pain.

This article was previously presented as a meeting abstract at the Las Vegas 2021 American College of Gastroenterology (ACG) Annual Scientific Meeting on October 25, 2021.

## Case presentation

**Figure 1:**
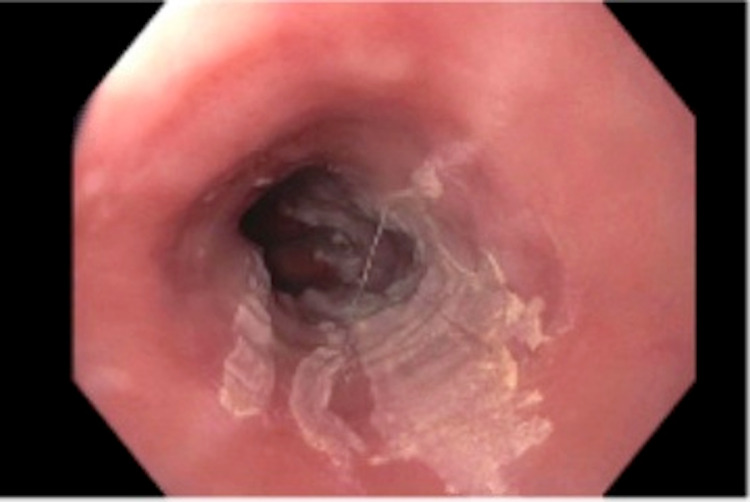
Endoscopy image showing esophageal mucosal sclerosis

A 54-year old male presented to the emergency room (ER) with a three-week history of sharp, non-radiating epigastric pain, nausea, and intractable non-bloody vomiting, which worsened over the last four days. He also reported anorexia and an unintentional 20-pound weight loss. He had no diarrhea, melena, hematochezia, dysphagia, odynophagia, chest pain, or dyspnea. He had a pertinent medical history of chronic arthritis treated with oral diclofenac. Initial vital signs were stable. Physical examination revealed severe dehydration with moderate epigastric tenderness. Notable labs included a blood urea nitrogen (BUN) of 18.5 mg/dl, Cr of 3.45 mg/dl, lipase of 132 U/l, but a normal complete blood count, liver panel, and serum lactate. A non-contrast computed tomography (CT) abdomen and pelvis was unrevealing. The patient's acute kidney injury resolved with intravenous (IV) fluids rehydration. Given his persistent symptoms and long-term oral diclofenac use, there was a concern for peptic ulcer disease (PUD). An upper gastrointestinal endoscopy (EGD) was done, which revealed a 2 cm hiatal hernia with esophageal mucosal sclerosis (Figure 1), gastritis, and congested duodenal mucosa.

**Figure 2:**
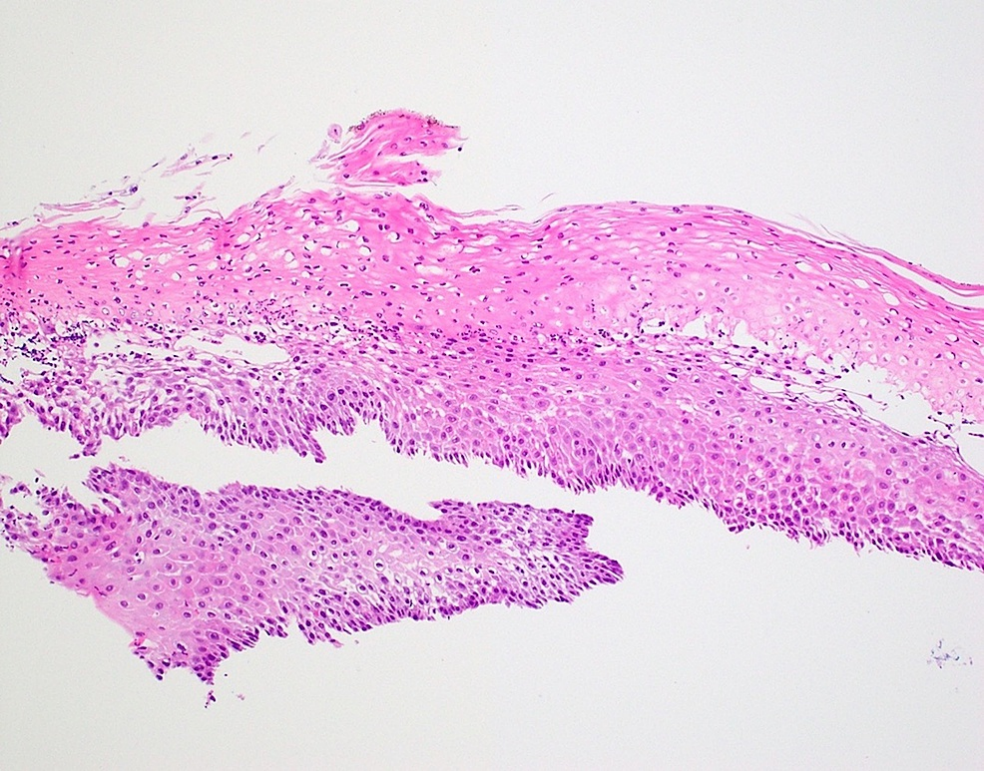
Histopathology section showing splitting of the squamous epithelium, a thick layer of parakeratosis at the surface, mild basal cell hyperplasia, and a mild intraepithelial inflammatory infiltrate composed predominantly of neutrophils

A distal esophageal biopsy confirmed EDS (Figure 2).

The oral diclofenac was discontinued, and he was treated with oral sucralfate and high-dose pantoprazole for a total of eight weeks; however, his symptoms completely resolved after only one week on therapy. He followed up with the outpatient gastroenterology clinic in six weeks with no complaints.

## Discussion

EDS is an infrequent benign condition of unclear etiology and pathogenesis. It has been linked to certain medications such as nonsteroidal anti-inflammatory drugs (NSAIDs), smoking, and autoimmune diseases. However, some cases remain idiopathic. While some patients are asymptomatic, others experience debilitating symptoms such as severe epigastric pain, dysphagia, abdominal pain, nausea, and vomiting [4]. Cases of patients coughing out large chunks of the sloughed mucosa have been reported [5]. The prevalence of EDS is higher among older, chronically debilitated patients on five or more medications [4,6]. EDS is endoscopically characterized by the sloughing of superficial strips of squamous mucosa into the esophageal lumen [7]. The histological analysis shows parakeratosis and splitting of the epithelial layers at different planes above the hyperplastic basal membrane. Minimal inflammation has been noticed despite EDS being termed “esophagitis” [1]. A retrospective study of 21497 EGD cases reported an EDS incidence of 0.03% [8]. The low incidence in combination with the typical contamination of biopsies with bacteria and fungi has led to frequent misdiagnosis and underreporting of EDS [1,3,9]. To date, there is no standardized therapy for EDS, as many of the cases are self-limiting with no long-term complications. Similar to our case, modification of the underlying EDS risk factors plus high-dose PPI use showed good results [4]. However, PPIs have been hypothesized to diminish further injury rather than treating the cause of EDS [10]. There is speculation that autoimmune-related EDS responds well to steroids, and Jaben et al. described an idiopathic case of EDS that responded well to steroids [5]. A follow-up endoscopy at eight weeks has been proposed by some authors but enough evidence does not yet support its benefit [9]. With the initiation of high-dose pantoprazole and stopping oral diclofenac, our patient had complete resolution of symptoms and was discharged home in good health.

## Conclusions

EDS remains a sporadic diagnosis that can affect the patient's quality of life. It is therefore important that clinicians appreciate the endoscopic appearance of EDS to avoid therapeutic delays. In addition, we highlight the importance of considering EDS as a differential while interpreting both endoscopic and histopathology results in correlation with the patient's clinical picture.
